# Characteristics of HIV-1 molecular transmission networks and drug resistance among men who have sex with men in Tianjin, China (2014–2018)

**DOI:** 10.1186/s12985-020-01441-8

**Published:** 2020-11-03

**Authors:** Minna Zheng, Maohe Yu, Shaohui Cheng, Ning Zhou, Tielin Ning, Long Li, Fangning Zhao, Xuan Zhao, Jingjin Zhu, Guohong Jiang

**Affiliations:** 1 Department for AIDS/STD Control and Prevention, Tianjin Centers for Disease Control and Prevention, No.6 Huayue Road, Hedong District, Tianjin, 300011 China; 2 Tianjin Centers for Disease Control and Prevention, No.6 Huayue Road, Hedong District, Tianjin, 300011 China

**Keywords:** HIV-1, Men who have sex with men, Transmission network, Tianjin, Drug resistance

## Abstract

**Background:**

In Tianjin, China, there is a relatively high prevalence of HIV in men who have sex with men (MSM). The number of HIV cases in Tianjin is also increasing. We investigated the HIV molecular transmission network, genetic tropisms, and drug resistance mutations in Tianjin.

**Methods:**

Blood samples were collected from 510 newly diagnosed antiretroviral therapy (ART)-naïve HIV-1-infected subjects among MSM in Tianjin. Partial *pol* and *env* genes were sequenced and used for phylogenetic, genetic tropism, and genotypic drug resistance analyses. Molecular clusters were identified with 1.5% genetic distance and 90% bootstrap support.

**Results:**

Among the 436 HIV-1 *pol* sequences obtained from the study participants, various genotypes were identified, including CRF01_AE (56.9%), CRF07_BC (27.8%), B (7.3%), CRF55_01B (4.1%), unique recombinant forms (URFs) (3.7%), and CRF59_01B (0.2%). A higher prevalence of X4 viruses was observed in individuals infected with CRF55_01B (56.3%) and CRF01_AE (46.2%) than with other subtypes. Of all 110 sequences in the 36 clusters, 62 (56.4%) were observed in 23 CRF01_AE clusters and 18 (16.4%) in four CRF07_BC clusters. Eight sequences clustered with at least one other shared the same drug resistance mutation (DRM). In different cluster sizes, the distributions of individuals by age, presence of sexually transmitted disease, and presence of DRMs, were significantly different.

**Conclusion:**

We revealed the characteristics of HIV molecular transmission, tropism, and DRMs of ART-naïve HIV-infected individuals among the MSM population in Tianjin. Identifying infected persons at risk of transmission is necessary for proposing counseling and treating these patients to reduce the risk of HIV transmission.

## Introduction

Since the first patient was diagnosed with acquired immunodeficiency syndrome (AIDS) in Beijing in 1985 [1], HIV-1 has evolved rapidly in China over 30 years, with an increasing number of infected individuals and increased genotype complexity [2, 3]. Sexual transmission has contributed greatly to the current HIV-1 epidemic in China. According to relevant reports, in 2017, the proportion of newly discovered HIV/AIDS cases in China through sexual transmission reached more than 90%, 20% of which were attributed to homosexual transmission among MSM [2–7]. Tianjin is one of the four direct-controlled municipalities in China, the gateway to Beijing, an important trading port, and neighbors Beijing and Hebei, with a total population of over 15 million people. Sexual transmission is the historically predominant route of HIV infection in Tianjin. In recent years, MSM transmission, as opposed to heterosexual transmission, has resulted in a tenfold increase in the total number of infections, particularly among young men in Tianjin [8]. The Tianjin Municipal Health and Family Planning Commission reported that MSM transmission cases accounted for 74.92% of all new diagnoses from January to October 2018, according to statistics from the AIDS prevention and control information management system. Phylogenetic analysis provides insight into the HIV transmission network structure. Typically, clusters are defined by the genetic distance between analyzed sequences and/or statistical support within the inferred tree [3]. Characterizing populations and forming transmission networks allow for targeted interventions to individuals at risk [4]. By identifying common clustering among MSM, we examined the HIV molecular transmission network and transmitted drug resistance mutations to determine the characteristics of the HIV epidemic in Tianjin at the provincial level to guide HIV prevention measures.

## Methods

### Study subjects

In total, 510 cases of newly diagnosed ART-naïve HIV infections were selected from the MSM population in Tianjin according to stratified random sampling after obtaining informed consent. Participants’ demographic data were obtained through face-to-face interviews prior to blood collection. Plasma (500 μL) was separated from whole blood within 24 h of collection and was used to determine the HIV-1 nucleotide sequences for subsequent analysis.

### HIV-1 RNA extraction, amplification, and sequencing

HIV-1 RNA was extracted from plasma using a QIAamp Viral RNA Mini kit (Qiagen, Hilden, Germany). Partial sequences of *pol* (HXB2: 2167–3440) and *env* (HXB2: 7022–7647) were amplified from extracted viral RNA [7, 9]. The *pol* and *env* fragments were amplified in a one-step reverse transcription polymerase chain reaction (RT-PCR) with primers using a Takara one-step RT-PCR kit (Shiga, Japan). Second-round PCR (nested PCR) was performed with primers using 2 × Taq PCR MasterMix (Takara) to increase the sensitivity and specificity of PCR. The primers and reaction cycling conditions are shown in Table 1. PCR products were identified by 1% agarose gel electrophoresis. Finally, positive products were sent to Anpu Biotechnology Company (Beijing, China) for sequencing.

**Table 1:** Primers and reaction cycling conditions for *pol* and *env*

Gene region	Primers		Reaction cycling conditions
*env*			
	ED5	5′-ATGGGATCAAAGCCTAAAGCCATGTG-3′ (sense)	RT-PCR: 50 °C 30 min 94 °C 2 min; 30 cycles of 94 °C 30 s, 55 °C 30 s, 72 °C 80 s; 72 °C 10 min
	ED12	5′-AGTGCTTCCTGCTGCTCCCA-3′ (anti-sense)	
	Env7a	5′-CTGTTAAATGGCAGTCTAGC-3′ (sense)	The second round: 94 °C 3 min, 30 cycles of 94 °C 30 s, 55 °C 30 s, 72 °C 80 s; 72 °C 10 min
	Env7a	5′-CTGTTAAATGGCAGTCTAGC-3′ (anti-sense)	
*pol*			
	MAW-26	5′-TGGAAATGTGGAAAGGAAGGAC-3′ (sense)	RT-PCR: 50 °C 30 min, 94 °C 2 min; 30 cycles of 94 °C 30 s, 55 °C 30 s, 72 °C 150 s; 72 °C 10 min
	RT-21	5′-CTGTATTTCTGCTATTAAGTCTTTTGA-3′ (anti-sense)	
	PRO-1^a^	5′-CAGAGCCAACAGCCCCACCA-3′ (sense)	The second round: 94 °C 5 min; 30 cycles of 94 °C 30 s, 63 °C 30 s, 72 °C 150 s; 72 °C 10 min
	RT-20^a^	5′-CTGCCAGTTCTAGCTCTGCTTC-3′ (anti-sense)	
	RT4R^a^	5′-CTTCTGTATATCATTGACAGTCCAGCT-3′ (anti-sense)	Sequencing
	RT1^a^	5′-CCAAAAGTTAAACAATGGCCATTGACAGA-3′ (sense)	Sequencing
	PROC1^a^	5′-GCTGGGTGTGGTATTCC-3′ (anti-sense)	Sequencing

^a^Sequencing primers

The obtained sequence fragments in the *pol* and *env* regions were edited and assembled using Sequencher 5.0 software (Gene Codes, Ann Arbor, MI, USA). The assembled sequences were aligned and checked manually together with the reference sequences retrieved from the Los Alamos HIV database (www.hiv.lanl.gov) in BioEdit 7.2.5 software. Duplicate sequences were eliminated using the ElimDupes network software from the Los Alamos National Laboratory HIV Sequence Database (https://www.hiv.lanl.gov/content/sequence/elimdupesv2/elimdupes.html).

### HIV-1 genotyping, drug resistance, and tropism analyses

HIV-1 subtypes were initially determined by neighbor joining phylogenetic analyses based on the Tamura-Nei model using MEGA X software with HIV-1 reference sequences. The significance of branch orders was tested by bootstrapping analysis with 1000 replicates. Recombination assessment employed two methods: a Simplot Bootscan (two-parameter Kimura model, window size 200 bp, 2-bp step) and Recombination Identification Program 3.0 (RIP 3.0, https://www.hiv.lanl.gov/content/sequence/RIP/RIP.html) [7, 9]. Additionally, to avoid potential errors, the sequences were compared with all known sequences in the HIV Database from Los Alamos National Laboratory, using the online HIV Basic Local Alignment Search Tool HIV Blast (https://www.hiv.lanl.gov/content/sequence/BASIC_BLAST/basic_blast.html). The genotype of each patient was determined based on the genotypes of both the *pol* and *env* regions. If only the *pol* region was available, the genotype of the region was determined.

The nucleotide sequences of* pol *gene, containing the full-length protease gene and the first 299 codons of the reverse transcriptase gene, were submitted to Stanford HIV Drug Resistance Database. HIV-1-transmitted drug resistance mutations (DRMs) were identified according to the WHO surveillance list for nucleoside reverse transcriptase inhibitors (NRTIs), non-nucleoside reverse transcriptase inhibitors (NNRTIs), and protease inhibitors (PIs) using the current Calibrated Population Resistance tool v5.0 (https://hivdb.stanford.edu/cpr/). The *env* sequences were analyzed for prediction of viral co-receptor usage based on *env* V3 loop sequences using the online tool Geno2pheno algorithm (https://coreceptor.geno2pheno.org) [10], with a false-positive rate cut-off of 10% according to European guidelines [8, 11].

### Phylogenetic and clustering analysis

We constructed the HIV *pol* genetic transmission network at a 1.5% distance threshold using Tamura-Nei93 (TN93) nucleotide substitution model in HIV-TRACE (www.hivtrace.org), and all sequences with pairwise distance ≤ 1.5% were identified. Phylogenetic and bootstrap analyses supporting branching with HIV-1 *pol* and *env* reference sequences were determined by the neighbor-joining method using the Tamura-Nei model with MEGA X, based on 1000 resamplings. Robust *pol* clusters were identified by combining the genetic distance in HIV-TRACE and bootstrap support values with 1000 replicates in MEGA X, which was consistent with the *env* clusters [12]. The codons associated with major DRMs defined by Lewis [13] were excluded to avoid the potential impact of convergent evolution [14].

### Statistics

Statistical comparisons were performed using Fisher’s exact and Chi^2^ tests for selected variables. Continuous variables were analyzed using the Mann–Whitney *U*-test for nonparametric statistics. Commercial software SPSS 24.0 (SPSS, Inc., Chicago, IL, USA) was used for statistical calculations. All tests were two-tailed, and values of *p* < 0.05 were considered statistically significant.

## Results

### Demographics and HIV genotyping of study subjects

HIV *pol* sequences were obtained for 436 of the 510 study subjects, with a success rate of 85.5%. HIV 384 *env* sequences were obtained for 436 subjects with *pol* sequences successfully amplified. The median age of these subjects was 29 years (range, 16–75): 94.0% (410/436) were Han ethnicity, 74.3% (324/436) were permanent residents, and 41.5% (181/436) had obtained college degrees or higher. Of these subjects, 66.5% (290/436) were single and 20.4% (89/436) were divorced or widowed. Of these subjects, 56.9% (248/436), 27.8% (121/436), 7.3% (32/436), 4.1% (18/436), and 0.2% (1/436) were infected with HIV genotypes CRF01_AE, CRF07_BC, B, CRF55_01B, and CRF59_01B, respectively. Additionally, 16 unique recombinant form (URF) samples (3.7%, 16/436) were observed with different genotypic identification from the *pol* and *env* regions (Table 2 and Fig. 2).

**Table 2:** Information on URF samples with different genotypic identification from *pol* and *env* regions

Sample ID	Year of diagnosis	Age (years)	Marriage	Ethnicity	Register	*pol* genotype	*env* genotype
16QR151	2016	22	Unmarried	Han	Tianjin	01BC	CRF01_AE
16QR673	2016	17	Unmarried	Han	Tianjin	01BC	CRF01_AE
16QR870	2016	24	Unmarried	Han	Other	CRF01_AE	CRF07_BC
16QRA20	2016	28	Unmarried	Han	Tianjin	CRF01_AE	CRF07_BC
16QRB97	2016	38	Unmarried	Han	Tianjin	01BC	CRF01_AE
17QR411	2017	46	Divorced/widowed	Han	Tianjin	01BC	CRF59_01B
17QR848	2017	27	Unmarried	Han	Other	01BC	CRF07_BC
17QR866	2017	45	Divorced/widowed	Han	Tianjin	01BC	CRF07_BC
17QRA14	2017	27	Unmarried	Han	Other	01BC	CRF07_BC
TJ150049	2015	25	Unmarried	Other	Tianjin	01BC	CRF07_BC
18LS1505	2018	24	Unmarried	Han	Other	01BC	CRF07_BC
18LS1657	2018	23	Unmarried	Han	Other	01BC	CRF07_BC
18LS4856	2018	36	Unmarried	Han	Tianjin	01BC	CRF07_BC
18LS5068	2018	22	Unmarried	Han	Tianjin	01BC	CRF07_BC
18LS5311	2018	21	Unmarried	Han	Tianjin	01BC	CRF07_BC
18LS5454	2018	24	Unmarried	Han	Tianjin	0107	01BC

### Characteristics of transmission clustering

**Fig. 1:**
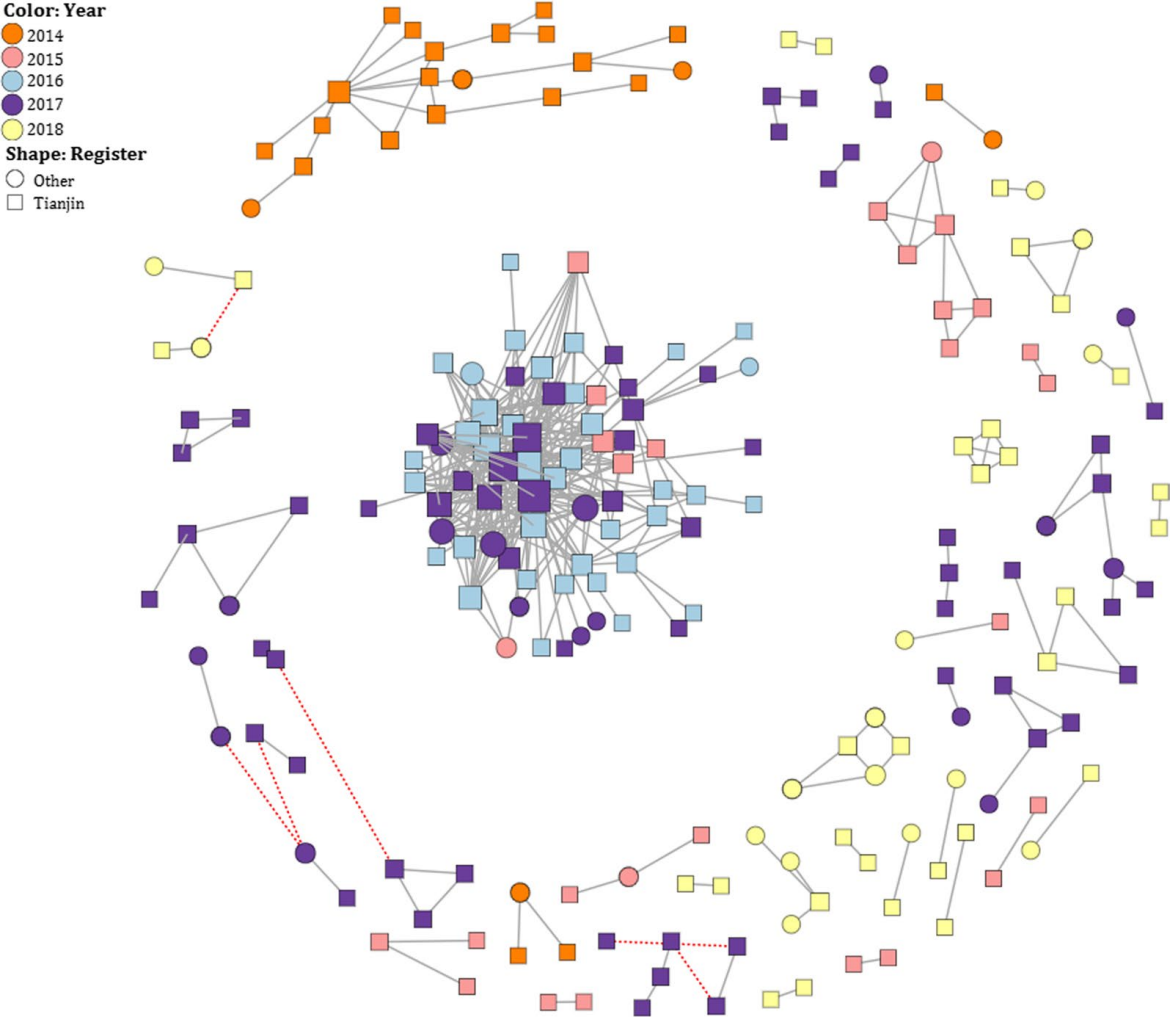
HIV transmission clusters *of pol* identified from newly diagnosed ART-naïve HIV infections in the MSM population in Tianjin, China (2014–2018) by HIV genetic transmission network using HIV-TRACE by a ≤ 1.5% genetic distance. Black lines indicate the worst-based support (p) of the length between the two nodes < 0.05. Red lines indicate the worst-based support (p) of the length between the two nodes ≥ 0.05

**Fig. 2:**
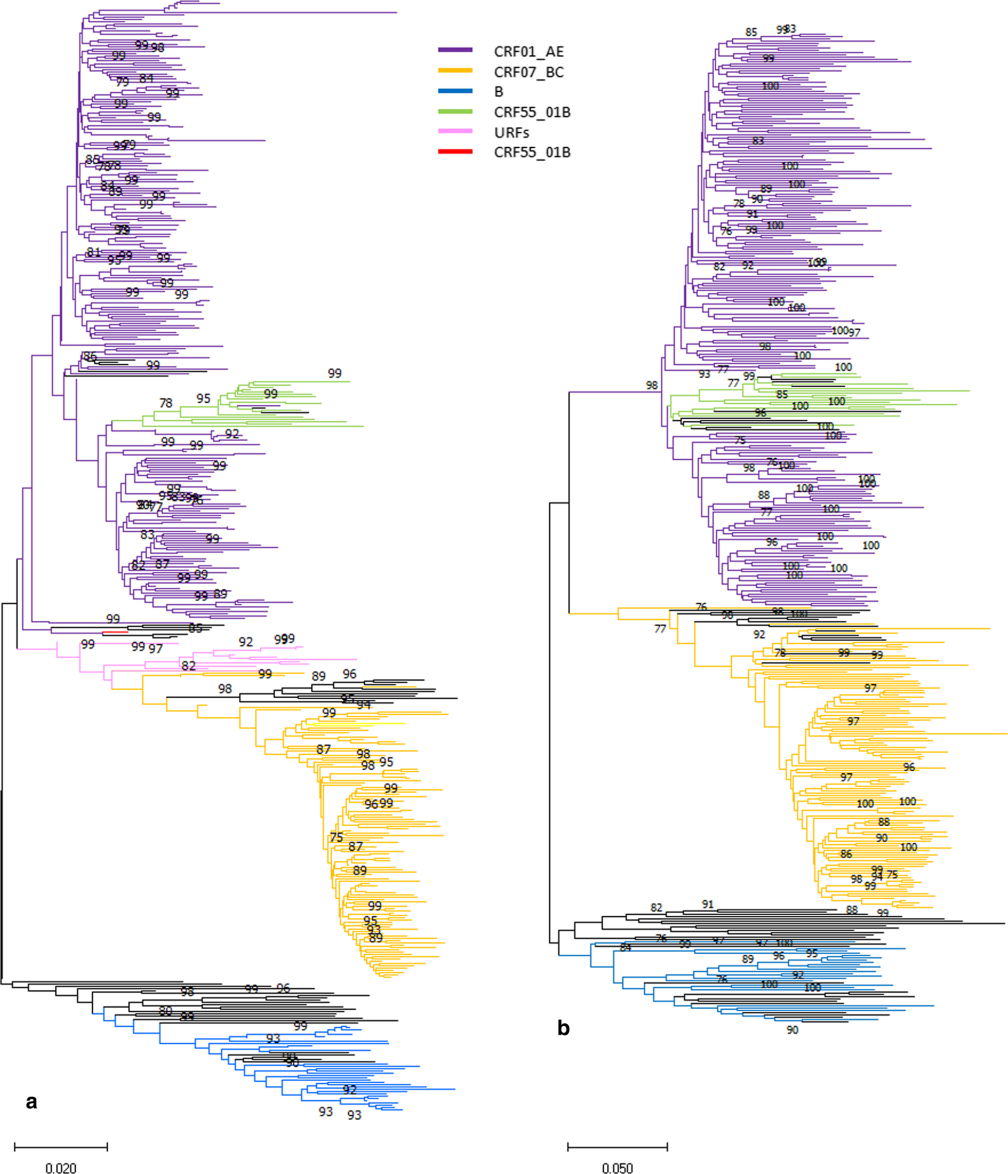
Phylogenetic tree analysis of partial *pol* (**a**) and *env* (**b**) region sequences from newly diagnosed ART-naïve HIV infections in the MSM population in Tianjin, China (2014–2018)

The *pol* sequences of 213 individuals were clustered in 42 clusters at 1.5% genetic distance threshold using the TN93 model in HIV-Trace (Fig. 1). Of the 213 *pol* sequences, 110 sequences in 34 clusters at bootstrap support values ≥ 90% in the neighbor-joining trees (Fig. 2a). Clustering performed based on partial *pol* was mostly sustained by the *env* sequences (Fig. 2b), except that two *pol* clusters were identified as 4 clusters because of two *env* clusters divided into two smaller clusters respectively. Thus, 36 robust clusters including 110 individuals (25.2%, 110/436) were identified according the clustering consistency analyzed and reference to relevant literature [15, 16]. Of the 110 clustered individuals in the 36 clusters, 78.2% (86/110) were in 32 small clusters (including 2–5 nodes) and 21.8% (24/110) were in four large clusters (including > 5 nodes) (Figs. 1, 2). The annual distribution of the individuals clustered showed an increasing trend from 2014 to 2016 followed by a decreasing trend (Table 3).

**Table 3:** Annual distribution of the individuals clustering and prevalence of DRMs

Year of diagnosis	Number of infections (%)^a^	Clustering (%)^a,b^	Prevalence of DRMs (%)^a,c^
2014	40 (100)	11 (27.5)	2 (5.0)
2015	57 (100)	16 (28.1)	2 (3.5)
2016	62 (100)	24 (38.7)	5 (8.1)
2017	158 (100)	35 (22.2)	14 (8.9)
2018	119 (100)	24 (20.2)	7 (5.9)
Total	436 (100)	110 (25.2)	30 (6.9)

^a^Numbers in parentheses indicate the proportion of HIV-1 cases as a percentage of each subgroup

^b^*χ*^*2*^ = 8.450, *p* = 0.075 calculated using Fisher’s exact method

^c^*χ*^*2*^ = 2.200, *p* = 0.697 calculated using Fisher’s exact method

### Co-receptor tropism

**Fig. 3:**
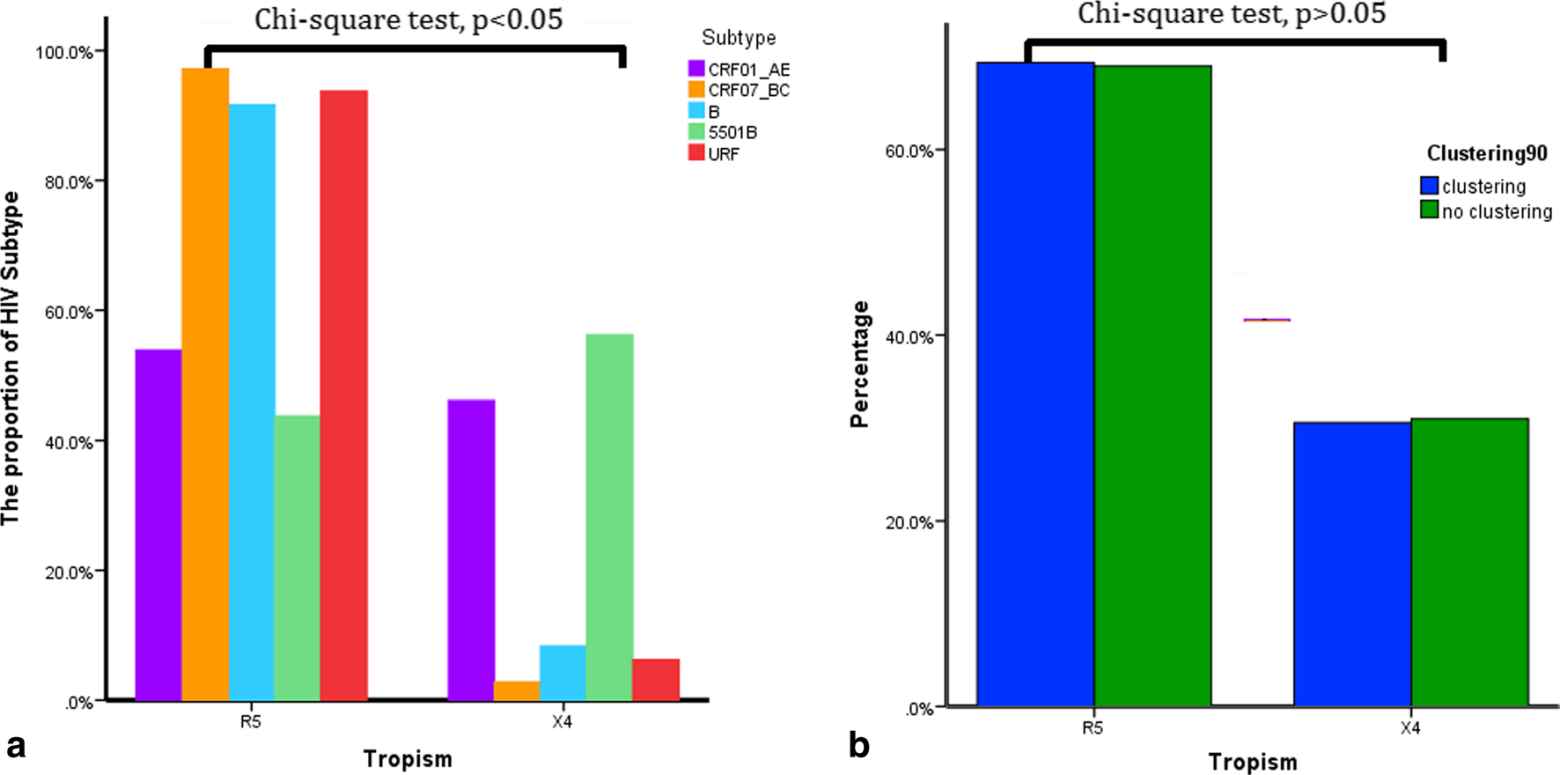
X4 and R5 co-receptor tropism of HIV 384 *env* sequences were obtained for 436 subjects with *pol* sequences successfully amplified. **a** Co-receptor usage based on different subtypes; **b** co-receptor usage based on clustering. *CRF* circulating recombinant form, *URFs* unique recombinant forms

Overall, of the 384 database-derived *env* V3 sequences, 117 (30.5%) were predicted as X4 tropisms and 267 (69.5%) as R5-tropic. As shown in Fig. 3, The prevalence of X4 viruses in individuals infected with CRF55_01B (56.3%, 9/16) and CRF01_AE (46.2%, 102/221) was higher than in those infected with CRF07_BC (2.8%, 3/107), subtype B (8.3%, 2/24), and URFs (6.3%,1/16) (*χ*^2^ = 92.839, *p* = 0.000). The proportion of R5-tropic viruses in the clusters was slightly higher than in no-clustering (69/98 = 70.4% vs 198/286 = 69.2%), and that of the X4-tropic viruses displayed the opposite trend, without significant differences (*χ*^2^ = 0.048, *p* = 0.899).

### Drug resistance (Table 3)

Among the 436 *pol* sequences in the ART-naïve population, 30 (6.9%) contained sequences harboring DRMs. The population prevalence rates of DRMs to NNRTI, NRTI, PI, and NNRTI + NRTI were 4.4% (19/436), 0.7% (3/436), 1.1% (5/436), and 0.7% (3/436), respectively. Furthermore, among the 19 sequences for resistance only to NNRTIs, 11 exhibited K101E mutations, four K103N mutations, one Y181C mutation, one Y188L mutation, and one V106M mutation, whereas one exhibited two mutations (K101E + K103N). The three sequences for resistance to NRTIs only exhibited the following mutation sites: D67N, K219E, and M184I. Among the 5 sequences for resistance to PIs, four sequences exhibited M46L and one I54T mutation. Of the three strains harboring multiple DRMs specific to both NRTIs and NNRTIs, one exhibited K70R + M184V + K103N + Y181C and two exhibited K219E + Y188C. The annual prevalence of DRM strains showed an increasing trend from 2014 to 2017 and then a decreasing trend from 2017 to 2018 (Table 3).

### Characterization of HIV molecular transmission networks

In the 32 small clusters (with 2–5 nodes), 56 nodes in 22 clusters were CRF01_AE subtypes, 12 nodes in three were CRF07_BC, eight nodes in three were 55_01B, eight nodes in three were URFs, and two nodes in one were B. Of the four large clusters, one including six nodes belonged to CRF07_BC, one cluster including six nodes belonged to CRF01_AE, and two clusters including six nodes belonged to B. For the different genotypes, the proportion of individuals involved in the networks exhibited significant differences **(**Table 4, *χ*^2^ = 62.667, *p* = 0.000). In the clusters with 2–5 nodes, the proportion of individuals infected with URFs (8/16 = 50%) was higher than in those infected with CRF55_01B (44.4%, 8/18), CRF01_AE (22.6%, 56/248), CRF07_BC (12/121 = 9.9%), subtype B (2/32 = 6.3%), and CRF59_01B (0/1 = 0.0%). In large clusters (with > 5 nodes), the proportion of individuals infected with subtype B (12/32 = 37.5%) was higher than in those infected with CRF07_BC (6/121 = 5.0%), CRF01_AE (6/248 = 2.4%), CRF55_01B (0/18 = 0.0%), URFs (0/16 = 0.0%), and CRF59_01B (0/1 = 0.0%).

**Table 4:** Characteristics of transmission clustered individuals

	Total N (%)^a^	Not clustered N (%)^a^	Clustered		*χ*^*2*^	*P*
			≤ 5 nodes/cluster N (%)^a^	> 5 nodes/cluster N (%)^a^		
Total (%)	436 (100)	326 (74.8)	86 (19.7)	24 (5.5)		
*Age (years)*					**113.777**	**0.028**^b^
< 25	108 (100)	73 (67.6)	30 (27.8)	5 (4.6)		
25–34	174 (100)	124 (71.3)	36 (20.7)	14 (8.0)		
35–44	70 (100)	59 (84.3)	10(14.3)	1(1.4)		
> 45	84 (100)	70 (83.3)	10 (11.9)	4 (4.8)		
*Marital status*					3.476	0.170
Single	290 (100)	209 (72.1)	65 (22.4)	16 (5.5)		
Married	57 (100)	47 (82.5)	8 (14.0)	2 (3.5)		
Divorced/widowed	89 (100)	70 (78.7)	13 (14.6)	6 (6.7)		
*Nationality*					4.465	0.314
Han	410 (100.0)	305 (74.3)	81 (19.8)	24 (5.9)		
Other	26 (100.0)	21 (80.8)	5 (19.2)	0 (0.0)		
*Education*					2.564	0.635
Middle school and below	127 (100.0)	89 (70.1)	31 (24.4)	7 (5.5)		
High school	128 (100.0)	99 (77.3)	22 (17.2)	7 (5.5)		
College and above	181 (100.0)	138 (76.2)	33 (18.2)	10 (5.5)		
*Permanent register*					1.610	0.447
Tianjin	324 (100.0)	237 (73.1)	68 (21.0)	19 (5.9)		
Other	112(100.0)	89 (79.5)	18 (16.1)	5 (4.5)		
*STDs*					**9.166**	**0.010**^b^
With	146 (100.0)	97 (66.4)	36 (24.7)	13 (8.9)		
Without	290 (100.0)	229 (79.0)	50 (17.2)	11 (3.8)		
*Genotype*					**62.667**	**0.000**^b^
CRF01_AE	248 (100.0)	186 (75.0)	56 (22.6)	6 (2.4)		
CRF07_BC	121 (100.0)	103 (85.1)	12 (9.9)	6 (5.0)		
B	32 (100.0)	18 (56.2)	2 (6.3)	12 (37.5)		
CRF55_01B	18 (100.0)	10 (55.6)	8 (44.4)	0 (0.0)		
URFs	16 (100.0)	8 (50.0)	8 (50.0)	0 (0.0)		
CRF59_01B	1 (100.0)	1 (100.0)	0 (0.0)	0 (0.0)		
*DRM*					**20.990**	**0.003**^b^
With	30 (100.0)	19 (63.3)	4 (13.4)	7 (23.3)		
Without	406 (100.0)	307 (75.6)	82 (20.2)	17 (4.2)		

^a^Numbers in parentheses indicate the proportion of HIV-1 subtypes as a percentage of each subgroup

^b^*p* < 0.05 calculated using Fisher’s exact method

The demographic characteristics of transmission clustered individuals are shown in Table 4. The age distribution of individuals in the different clustering patterns showed significant differences (*χ*^2^ = 113.7777, *p* = 0.028). In the large clusters, the proportion of individuals in the 25–34 year age group (14/174, 8.0%) was higher than those in other age groups. In small clusters, the proportion of individuals in the < 25 years age group (30/108, 27.8%) was higher than those in the other age groups. There was no difference in the marital status, nationality, residence characteristics, and education characteristics of individuals in the different clustering patterns (Table 4). Of the 36 transmission clusters in the network, 17 transmission clusters contained 23 individuals who were non-Tianjin (including eight cases were registered in Hebei province, four in Jilin, four in Heilongjiang, three in Anhui, two in Shandong, and one in Shanxi and Sichuan, respectively) (Fig. 1).

The proportion of individuals with sexually transmitted diseases (STDs) (8.9% and 24.7%) was higher than the proportion of individuals without STDs (3.8% and 17.2%) in both large and small clusters (Table 4, *χ*^2^ = 9.166, *p* = 0.010). The proportion of individuals with DRM (7/30, 23.3%) was higher than that of individuals without DRM (17/406, 4.2%) in the large clusters (Table 4, *χ*^2^ = 20.990, *p* = 0.003). Of the 11 clustered sequences with at least one DRM, eight clustered with at least one other shared the same DRM. One of the three strains harboring multiple DRMs to both NRTIs and NNRTIs (K70R + M184V + K103N + Y181C) was confirmed to be clustered in the genetic transmission networks.

## Discussion

Based on phylogenetic and demographic parameters, we analyzed nucleotide sequences for 436 newly diagnosed patients among MSM to track the characteristics of HIV-1 transmission networks. The results revealed an epidemic characterized by high heterogeneity in the subtypes and high prevalence of recombinant forms of infection (92.7%). From the results of our local MSM cohort, since 2014 CRF55_01B strains were discovered successively and the type and number of recombinant genotypes are increasing, leading the more complicated epidemic trends. Recombination is an important mechanism contributing to the genetic diversity of HIV-1 [16]. Thus, an increasing number of circulating recombinant forms (CRFs) and URFs have been reported on a global scale [17, 18]. A total of 102 HIV-1 CRFs are listed in the Los Alamos National Laboratory HIV database (https://www.hiv.lanl.gov/content/sequence/HIV/CRFs/CRFs.html). The emergence of novel recombinant forms may easily occur via co-circulation and dual infection of multiple HIV-1 genotypes among MSM in Tianjin, such as subtype B, CRF01_AE, and CRF07_BC. The recombinant forms are increasing the complexity of the HIV-1 epidemic among the MSM cohort in Tianjin. Therefore, effective HIV-1 molecular epidemiologic investigations are needed to identify the transmission of potential HIV-1 recombinant forms in Tianjin, China [9].

HIV transmission clusters are most often identified by phylogenetic analysis based on similarities in viral sequences [19, 20]. Cluster inclusion thresholds tend to be ad hoc, and there is no widely accepted definition [20]. Comparison of the results from several previous studies can be confounded by varying populations, sampling fraction, individual risk profiles, and varying methods used for cluster identification [21]. Traditionally, statistical node support for the relationships in a phylogenetic tree is evaluated by bootstrapping [22]. Different studies have used bootstraps ranging from 70 to 99% in combination with genetic distances of 1% ± 4.5%, more than 90% indicating strong support for a group [23]. In a study of local and national HIV surveillance in the USA, a *pol* genetic distance of two individuals of ≤ 1.5% implies a direct or indirect epidemiological linkage [24]. Previous studies revealed *pol* mean estimated evolutionary rates for CRF01_AE, CRF07_BC, and B of 2.54–2.97 × 10^–3^, 1.71–2.03 × 10^–3^, and 2.09 × 10^–3^ substitutions/site/year in China [5–7]. For this study, we used strict criteria to identify all clusters based on a mean genetic distance of ≤ 1.5% and bootstrap value ≥ 90%, considered the sampling fraction, methodology, and convenience of follow-up. In this analysis, we found that 25.2% of all newly diagnosed cases among MSMs were included in 36 transmission clusters. In the different sized clusters, the CRF55_01B, CRF01_AE, and CRF07_BC (6/18) viruses were mostly in small clusters, whereas B viruses were mostly in large clusters. The clustering characteristics of different subtypes may be related to strain variation and/or lack of linkages because of sample density [5–7]. However, individuals with more linkages may have a higher transmission risk [5–7]. Thus, intra-group concentrated transmission of different subtypes requires further analysis. From 2014 to 2018, the annual trends in the individuals clustered may be associated with current treatment strategies in China and/or lack of some lineages caused by sample selection bias. In 2014, the standard of free antiviral treatment for AIDS in China was adjusted from 350 to 500 CD4-cells/µL. In 2016, the standard was further adjusted to "Discovery is Treatment". Our study in Tianjin also revealed a decreased annual prevalence of DRMs in 2018 after an overall increasing from 2014 to 2017 (Table 2). Whether universal free access to medical care and ART for more patients can reduce further transmission should be evaluated in longer surveillance studies.

In China, the regimen composed of TDF, 3TC, and EFV is currently the most commonly used free first-line therapy [25]. Our study highlights that DRMs affecting the efficacy of NNRTIs are the most common, followed by those of NRTIs and PIs, which is consistent with results of domestic and foreign studies [25–27]. Related studies in the USA showed that the K103N mutation was the most common, and its generation and transmission were related to the failure of early antiviral therapy caused by patients' long-term and frequent use of the NNRTI drug efavirenz [26]. In our study, K101E was the most frequently observed mutation in response to NNRTIs, followed by K103N. Whether this is related to region, race or the extensive application of NNRTI drugs and early antiviral failure in antiviral therapy in China should be further evaluated. However, our study showed that the transmission of viruses containing DRMs exhibited the significant increase in large clustered infections (Table 4), and one of the three strains harboring mutations responsible for drug resistance to NRTIs and NNRTIs were confirmed to be clustered in the genetic transmission networks. As early as 2007, a study by Art et al. showed that approximately half of transmitted resistance can be attributed to clustered infections [28]. Because of the increase in the prevalence of drug resistance and emergence of multi-resistant mutant strains, DRM surveillance is necessary to prevent the spread of HIV.

Since the CCR5 blocker maraviroc was applied clinically for treating patients extensively harboring R5 viruses in Europe and America, studies have focused on HIV-1 tropism [11]. Nevertheless, current HIV-1 co-receptor usage in China has not been fully characterized. Understanding the co-receptor usage of HIV strains is essential for assessing the candidacy of CCR5 antagonists for treating HIV infection in China [29, 30]. Simultaneously binding to CD4 and two main co-receptors, CCR5 or CXCR4, is a necessary condition for HIV to infect target cells. Co-receptor selectivity is determined by genetic sequences within the HIV *env* “V3” region, which is involved in co-receptor binding [30]. HIV-1 variants are classified as R5- and X4-tropic viruses according to the ability to use CCR5 or CXCR4, respectively. The CCR5 antagonists, which inhibit HIV-1 binding on the CCR5-coreceptor, are only active on R5-tropic viruses, indicating tropism determination before prescription. Therefore, the determination of tropism is useful in clinical practice [31]. Several studies have supported the prevalence of tropism associated with HIV disease progression [32–34]. R5-tropic viruses are predominant in the early stages of HIV infection, because of preferentially selective transmission for the viruses as a biological bottleneck inherent to the genital mucosa [34]. We found that the proportion of R5-tropic virus clustering is slightly higher than that of non-clustering without significant differences. In addition, several studies have shown that X4-tropism for CRF01_AE recombinant is associated with accelerated progression to AIDS [32–34]. We observed a high prevalence of CRF01_AE and CRF55_01B X4 strains. CRF01_AE is the most prevalent in China and has contributed to 84% of HIV infections in Asia [32–34]. Further transmission of X4-tropic CRF01_AE and its second-generation recombinant strains may impede treatment with CCR5 antagonists in the future. Various antagonists are urgently required for effective and targeted treatment in this situation.

By utilizing the information from the inferred molecular HIV transmission network, we combined the demographic, clinical, and molecular data and found that 33.6% of individuals with STDs appeared in the clusters. This is consistent with recent reports on the increasing coincidence of HIV with STDs and high-risk sexual behaviors [35]. Seventeen of the 36 transmission clusters in the network contained 23 individuals with non-Tianjin permanent register. These individuals were primarily from northern Chinese provinces, including Hebei, Jilin, and Heilongjiang. Therefore, measures of the prevention and control of HIV transmission between Tianjin and major provinces are needed.

## Conclusion

Our study illustrated the characteristics of HIV molecular transmission, tropism, and drug resistance of ART-naïve HIV infections among the MSM population in Tianjin. It is necessary to determine which individuals of a population are at an increased risk of infection to intervene before further transmission occurs and to administer appropriate treatments. Moreover, the cooperation between Tianjin and neighboring provinces regarding HIV prevention and control should be strengthened. Based on the prevalence of tropism, we suggest that tropism testing of the HIV-1 V3 gene is pivotal for controlling transmission and treatment of HIV infections in China.

## Data Availability

The datasets used and/or analyzed during the current study are available from the corresponding author on reasonable request.

## Abbreviations

MSM: Men who have sex with men
ART: Antiretroviral therapy
AIDS: Acquired immunodeficiency syndrome
DRM: Drug resistance mutation
NRTIs: Nucleoside reverse transcriptase inhibitors
NNRTIs: Non-nucleoside reverse transcriptase inhibitors
PIs: Protease inhibitors
FPR: False-positive rate
URFs: Unique recombinant forms
STDs: Sexually transmitted diseases
CRFs: Circulating recombinant forms
